# Breakfast Characteristics and Its Association with Daily Micronutrients Intake in Children and Adolescents–A Systematic Review and Meta-Analysis

**DOI:** 10.3390/nu12103201

**Published:** 2020-10-20

**Authors:** Natalia Giménez-Legarre, María L. Miguel-Berges, Paloma Flores-Barrantes, Alba M. Santaliestra-Pasías, Luis A. Moreno

**Affiliations:** 1 GENUD (Growth, Exercise, Nutrition and Development) Research Group, Facultad de Ciencias de la Salud, Universidad de Zaragoza, 50009 Zaragoza, Spain; mlmiguel@unizar.es (M.L.M.-B.); pfloba@unizar.es (P.F.-B.); albasant@unizar.es (A.M.S.-P.); lmoreno@unizar.es (L.A.M.); 2 Instituto Agroalimentario de Aragón (IA2), 50013 Zaragoza, Spain; 3 Instituto de Investigación Sanitaria Aragón (IIS Aragón), 50009 Zaragoza, Spain; 4 Centro de Investigación Biomédica en Red de Fisiopatología de la Obesidad y Nutrición (CIBERObn), Instituto de Salud Carlos III, 28029 Madrid, Spain

**Keywords:** breakfast, nutrient, micronutrient intake, children, adolescents

## Abstract

Breakfast is an important source of key nutrients in the diet. For this reason, the aim of this review was to investigate the associations between breakfast consumption and daily micronutrients intake in both children and adolescents (aged 2–18 years). A peer-reviewed systematic search was conducted in three datasets (PubMed, Scopus and Cochrane Library) in February 2020 in English and Spanish. Two independent reviewers evaluated 3188 studies considering the AXIS critical appraisal and PRISMA methodologies. Meta-analysis was carried out comparing results according to type of breakfast consumed (Ready to eat cereals (RTEC) breakfast or other types of breakfast) and breakfast skipping. Thirty-three articles were included in the systematic review (SR) and 7 in the meta-analysis. In the SR, we observed that those children and adolescents who usually consume RTEC at breakfast had a higher consumption of B-vitamins than those not consuming RTEC at breakfast. Breakfast consumers had a higher mineral intake (iron, calcium, magnesium, potassium, zinc, and iodine) than breakfast skippers. In the Meta-Analysis, RTEC consumers had significantly higher vitamin C intake than breakfast skippers (Standard Mean Difference (SMD), −4.12; 95% confidence intervals (CI): −5.09, −3.16). Furthermore, those children who usually consume breakfast had significantly higher daily intake of calcium than breakfast skippers (SMD, −7.03; 95%CI: −9.02, −5.04). Our review proposes that breakfast consumption seems to be associated with higher daily micronutrients intake than breakfast skippers.

## 1. Introduction

Breakfast is extensively recognized as an important component of a healthy lifestyle and represents an important source of key nutrients in the diet for both adults and young population groups [1,2,3,4,5], showing several benefits of its consumption [2,3,4]. The International Breakfast Research Initiative (IBRI), developed in six countries, observed that there was a general breakfast pattern. In children, the proportion of breakfast consumers was very high (from 87.4% in US to 98.9% in Denmark) but declined with adolescence (from 74.2% in US to 87.9% in Canada) [6].

Breakfast consumption showed a protective effect against obesity and overweight [7]; for this reason, breakfast consumption is important because of its inverse association with body fat [8,9]. For instance, previous authors observed that children with obesity were more likely to skip breakfast [9,10,11], and these children had a high risk of chronic diseases as type 2 diabetes [12,13,14], dyslipidemia or cardiovascular diseases [7,8,15]. Likewise, previous authors observed that some vitamin deficiencies could be associated with fatness and other diseases, [16,17,18] and can be considered a critical public health issue [19,20]. Some studies also observed that in both children and adolescents, breakfast consumption has a positive effect on cognitive performance [21]. Nevertheless, breakfast is the meal that they most frequently skipped [22,23].

It has been suggested that children should consume about 20% of their daily energy intake (EI) at breakfast [24,25,26]. Different studies showed that those who often skip breakfast had lower EI and macronutrient’s intake compared with those who usually consumed breakfast [16,27,28,29]. Breakfast consumption has been associated with a better intake of vitamins and minerals in both children and adolescents [30]. In the same way, it has been observed that those adolescents who usually consume breakfast are more likely to meet recommended intakes of some minerals such as iron, calcium, zinc, magnesium, and copper, and vitamins A, B_6_, B_1_, C, D, E, and folate [31]. However, no systematic analysis of these associations was previously performed.

Nowadays, ready-to-eat-cereals (RTEC) at breakfast have become increasingly popular due to the desire for an easy alternative [32]. RTEC are increasingly consumed, and children and adolescents prefer RTEC instead of other cereal-based foods, such as bread [33,34]. In a previous Spanish study, RTEC consumption was associated with a low risk for inadequate micronutrients intake in children, adolescents and young adults [35].

To the authors’ knowledge, this is the first systematic review (SR) regarding the association between breakfast consumption and daily micronutrients intake. However, a recent SR and meta-analysis observed that breakfast consumption is associated with healthier food and beverages consumption and also better macronutrient intake [36].

The aim of this SR is to observe the associations between characteristics and frequency of breakfast consumption and total daily diet composition in terms of micronutrients intake.

## 2. Methodology

### 2.1. Protocol

This SR is the follow up of a previous SR that has been published [36]. Both SR shared the same methodology, including different studies taking into consideration the specific aims of each one. This SR has been performed following the criteria and methodology established by the Preferred Reporting Items for Systematic reviews and Meta-Analyses Protocols (PRISMA) [37]. The SR was registered in ‘Prospero’ CRD42020142570. Using the PICO principle (Participants, Intervention, Control, Outcomes), a specific question was constructed (Table 1) [38].

### 2.2. Search Strategy

Journal articles were identified by searching in electronic databases and scanning references and lists of articles. The search strategy was applied to PubMed, Scopus and the Cochrane Library databases in February 2020. The search strategy used to identify the article was as follows: (“Breakfast”(Mesh) OR “Breakfast”(tiab)) AND (“Food”(Mesh) OR “Beverages”(Mesh) OR “Diet, Food and Nutrition”(Mesh) OR “Diet”(Mesh) OR “Eating”(Mesh) OR “Feeding Behavior”(Mesh) OR “Nutritional Requirements”(Mesh) OR “Nutritional Status”(Mesh) OR “Nutritive Value”(Mesh) OR “breakfast skipping” (tiab) OR “meal Skipping”(tiab) OR “Fasting”(Mesh) OR “Food preferences”(Mesh) OR “Diet therapy”(Mesh) OR “Energy Intake”(Mesh) OR “nutrient”(Mesh)) AND (“Child, Preschool”(Mesh) OR “Child”(Mesh) OR “Adolescent”(Mesh) OR “breakfast skipping” OR “meal skipping”).

### 2.3. Selection Criteria

The following inclusion criteria were used: (1) types of studies: cross-sectional, longitudinal, descriptive study or case control study, (2) participants between 2–18 years, (3) original studies in which assessment of breakfast has been done/performed, and (4) publications in English and Spanish.

All articles were collected into an online citation manager (EndNote® Online, EndNote X9.1, Clarivate Analytics) and were screened for potential relevance according to title and abstract based on inclusion and exclusion criteria. The full text of all those studies fulfilling the inclusion criteria were collected and were evaluated for relevance according to the aim of the SR. In doubtful cases, studies were discussed with the SR team. Detailed information regarding publication’s year, study design, setting, participants, and results on micronutrient outcomes were extracted. The literature search was not limited by any range of years.

### 2.4. Systematic Review Process and Data Extraction, and Synthesis

**Figure 1:**
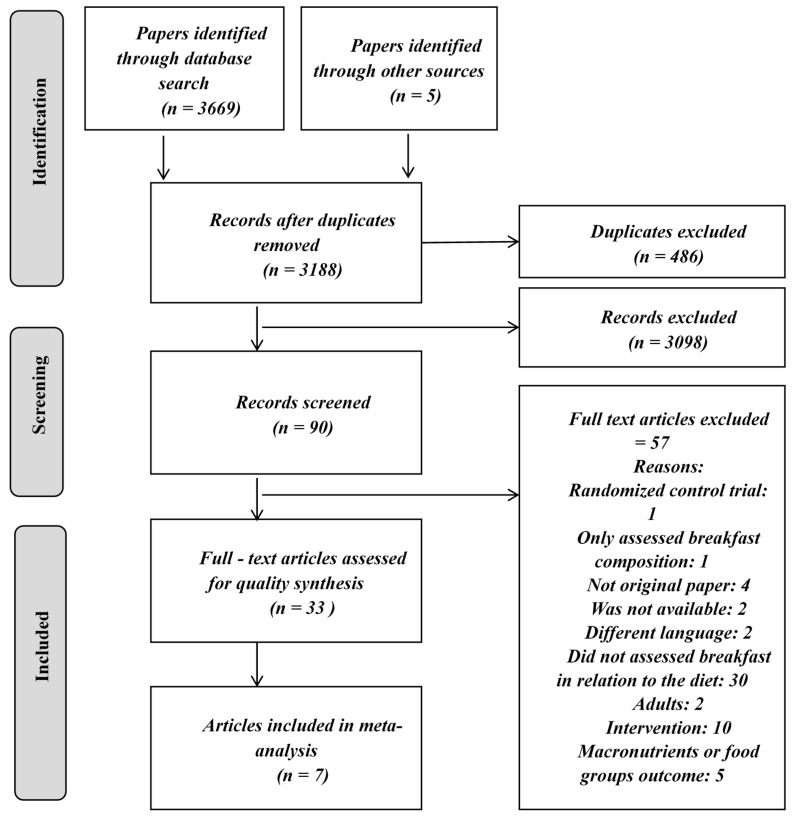
Flowchart diagram of study selection and search.

Two reviewers independently (NGL and PFB) evaluated all studies. Titles and abstracts were examined, and full relevant articles were obtained and assessed using the mentioned inclusion and exclusion criteria. Inter-reviewer disagreements were resolved by consensus, and in some cases, a third reviewer was consulted to solve disagreements. Figure 1 shows in a flowchart the search results.

### 2.5. Quality and Risk of Bias Assessment

The Appraisal tool for Cross-sectional Studies (AXIS) was used to assess the risk of bias and the methodological quality of included studies [39]. The tool consists of a 20-question-list, which included the study design, target population, sample size and selection, sampling frame, reliability and validity of the measurements, methodology, ethical issues, and conflict of interest. The details of the assessment of each included study are shown in the supplementary section, Table S1. Given that the tool does not provide an accumulated score on quality, the results have been summarized on Table 2 in order to obtain a general view of the quality of all the included studies.

### 2.6. Statistical Analyses

All analyses were performed using Open Meta (Analyst) software (open-source, cross-platform software for advanced meta-analyst). Two possibilities for comparison groups were assessed: RTEC breakfast versus skip breakfast, and other types of breakfast versus skip breakfast. Mean difference (MD) with 95% confidence intervals (95%CI) was used for comparing micronutrients (in milligrams (mg) or micrograms (μg)) between skip breakfast, RTEC breakfast and other types of breakfast. Also, for continuous data, random effects models with DerSimonian and Laird values were applied. For each outcome, effect sizes were calculated.

When information was available, the I2 statistic and its associated *p* value was used to test the heterogeneity, which describes as a proportion of the total variance the variance between studies [40]. Low heterogeneity was indicated with a value of <25%; high heterogeneity was indicated with a value of >50% to 75%, and high heterogeneity was indicated with a value of >75%. The absence of heterogeneity was determined with a non-significant *p* value.

## 3. Results

### 3.1. Literature Search and Screening

The included studies are summarized in Figure 1. A total of 3674 eligible papers were identified: 2544 from PubMed, 620 from Scopus, 505 from the Cochrane Library, and 5 were identified through other sources (Figure 1).

After duplicates exclusion and title reading, 3188 articles were assessed for eligibility. Finally, only 33 full-text articles met the inclusion criteria and 7 of them were considered for meta-analysis.

### 3.2. Study Design Characteristics

All the included articles were published in English and Spanish. From the 33 included articles, only 3 articles (9.1%) were longitudinal versus 90.9% of the studies (*n* = 30), which were cross-sectional. From the 33 included studies, 17 were performed in representative samples of the correspondent population [16,28,41,42,43,44,45,46,47,48,49,50,51,52,53,54,55]. Table 3 shows details of the articles, which included the impact of breakfast consumption on micronutrients intake. The studies were conducted between 1981–2018 in the following countries: United States (US) (*n* = 11) [30,41,44,49,51,52,55,56,57,58,59], United Kingdom (UK) (*n* = 5) [3,29,47,60,61], Canada (*n* = 3) [42,43,54], Spain (*n* = 2) [62,63], Australia (*n* = 3) [45,46,53], México (*n* = 1) [28], Ireland (*n* = 1) [64], Belgium (*n* = 1) [65], Cyprus (*n* = 1) [66], France (*n* = 1) [50], Malaysia (*n* = 1) [67], Japan (*n* = 1) [68], and 3 were combined European countries [16,48,66]. Data came from three sources: most of them were national health studies (60.6%) [3,28,30,41,42,43,44,45,46,47,51,52,53,54,55,56,57,59,61,68], 30.3% were original studies [29,49,50,58,60,62,63,64,65,67] and the rest were European multi-center studies (9.0%). Results of the studies have been differentiated according to the age of the participants, ranging from 2–12 years for children, and from 13–18 years for adolescents. Included studies were performed in children 39.3% (*n* = 13) [28,29,47,49,51,52,56,57,58,62,63,66,67], 12.1% (*n* = 4) adolescents [16,48,64,65], and both children and adolescents 48.4% (*n* = 16) [3,30,41,42,43,44,45,46,50,53,54,55,59,60,61,68].

### 3.3. Reporting Methods

Results are presented in Table 3 in alphabetic order per author’s last name.

Outcome variables were analyzed using 24 h Dietary Recalls (24 h-DR) (*n* = 26) or Food Records (FR) (*n* = 8). Two studies assessed diet with both 24 h-DR and Food Frequency Questionnaire (FFQ) questionnaires. Fulfilling of questionnaires was dependent on the methodology, the questionnaires used and on the age of the participants (interview with parents or caregivers, reported by parents or caregivers or self-reported by the children or adolescents).

Several articles assessing the consumption of RTEC were included (*n* = 22, 66.6%). Frequency and quantity consumed per week were assessed as well as the association of its consumption with different behaviors and health outcomes. Comparison groups according to breakfast consumption and/or type of breakfast consumed were the following: (*n* = 8) ‘frequency of consumption of RTEC’ [29,47,49,56,58,61,64,69], (*n* = 8) ‘RTEC consumers versus non-RTEC consumers’ [46,48,50,54,57,59,62,67], (*n* = 5) ‘breakfast skippers versus breakfast consumers versus RTEC consumers’ [41,42,44,45,52], (*n* = 8) ‘frequency of breakfast consumption (breakfast skippers versus breakfast consumers) [3,16,30,43,46,51,53,68], (*n* = 3) comparisons between different types of breakfast [28,55,66], (*n* = 2) comparisons between nutritional composition of breakfast [50,63], and one of them considered breakfast quality [65]. Two studies had two comparison groups according to breakfast consumption.

Of the relevant studies (*n* = 30), 90.9% showed associations between breakfast consumption and daily vitamins intake. Breakfast consumption and vitamin C intake was the most evaluated relationship in the vitamins group, which was analyzed in 27 studies; from which 15 showed significant associations.

Breakfast consumption and B vitamins’ associations were widely analyzed and in most of the studies, positive significant associations were found. Thiamin intake was found to be positively associated with breakfast consumption in 19 out of 22 studies, riboflavin in 19 out of 22, niacin in 12 out of 20, pyridoxine in 16 out of 20, folate in 18 out of 22, and cobalamin in 11 out of 15. Pantothenic acid (vitamin B5) intake was only assessed in two studies, from which positive and significant associations with breakfast consumption were also found.

Vitamin A and vitamin D were positively associated with breakfast consumption in 12 studies. On the other hand, intake of vitamin E was evaluated in 11 studies and most of them (9 studies) did not find significant associations. Vitamin K was evaluated in four studies, and in three of them the authors did not find significant associations.

Breakfast consumption and mineral dietary intakes were assessed in 32 studies. The association between breakfast consumption and calcium intake was the most frequent relationship, evaluated in 31 studies in the minerals group. Positive associations were found between breakfast consumption and iron intake (26/29), sodium (11/20) and zinc (14/18). Iodine was evaluated in two studies and both showed significant associations. Magnesium and potassium intakes were positively associated with breakfast consumption in 15 studies. Regarding phosphorous, only one author found significant associations (1/12). However, every study found significant associations between breakfast consumption and copper and manganese intake.

### 3.4. Meta-Analysis Results: Measurement of the Effect of the Relationship between Type of Breakfast and Micronutrients Intake

**Figure 2:**
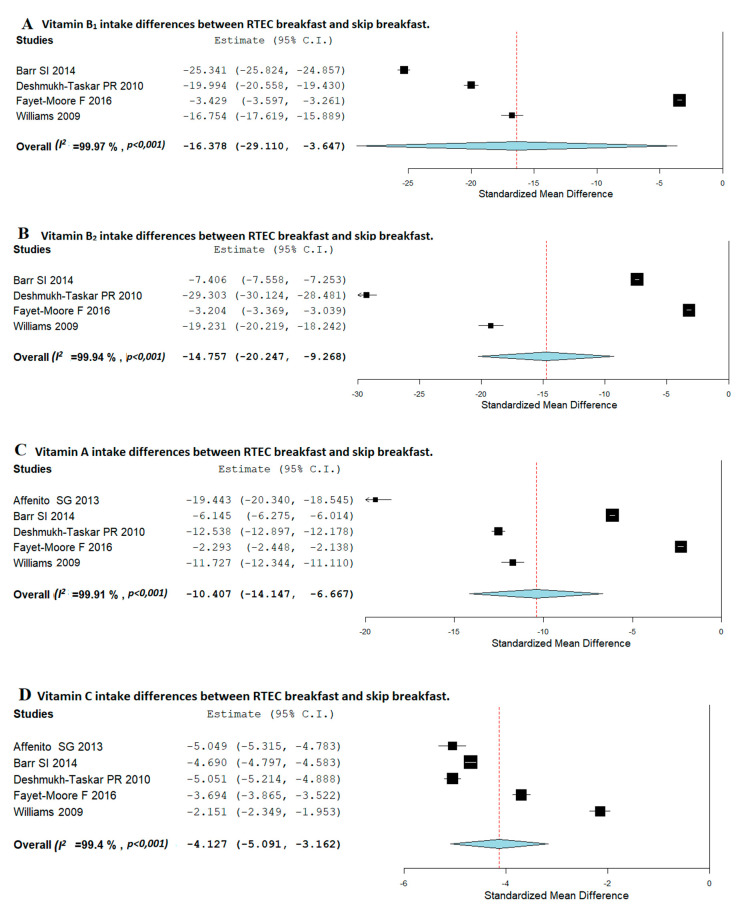
Random-effects meta-analysis of the effects of relationships between Ready to Eat Cereal (RTEC) breakfast and breakfast-skipping concerning vitamin B1 (**A**), vitamin B2 (**B**), vitamin A (**C**), and vitamin C (**D**) intake. CI: confidence intervals.

**Figure 3:**
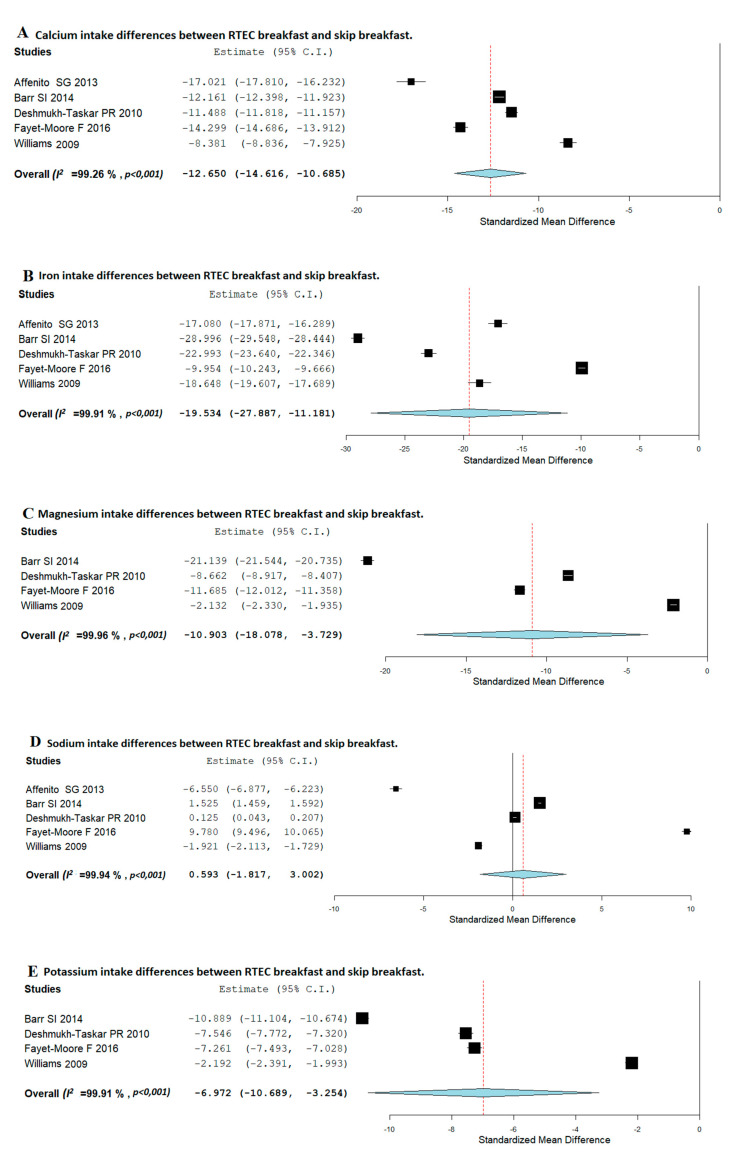
Random-effects meta-analysis of the effects of relationships between RTEC breakfast and breakfast-skipping concerning calcium (**A**), iron (**B**), magnesium (**C**), sodium (**D**), and potassium (**E**) intake.

Differences in vitamins and minerals consumption between RTEC breakfast consumers and breakfast skippers are shown in Figure 2 (vitamins) and Figure 3 (minerals).

Regarding vitamins intake, as presented in Figure 2A, in children, RTEC consumers had significantly higher daily consumption of thiamine (vitamin B1) than children who usually skip breakfast (SMD, −16.378; 95%CI: −29.110, −3.647). However, heterogeneity amongst studies was high (*I*^2^ = 99.97%; *p* < 0.001). Figure 2B shows that RTEC consumers had significantly higher daily intake of riboflavin (vitamin B2) than children who usually skip breakfast (SMD, −14.757; 95%CI: −20.247, −9.268). Heterogeneity amongst studies was very high (*I*^2^ = 99.94; *p* < 0.001). Analyzing the consumption of Vitamins A and C, Figure 2C,D shows that those children who usually consume RTEC had a significantly higher intake in both vitamins than those who usually skip breakfast (SMD, −10.407; 95%CI: −14.147, −6.667 and SMD, −4.127; 95%CI: −5.091, −3.162), respectively, the heterogeneity amongst studies being very high (*I*^2^ = 99.91; *p* < 0.001 and *I*^2^ = 99.4; *p* < 0.001, respectively).

Regarding minerals consumption, Figure 3A,B shows that children who usually ate RTEC breakfast had significantly higher daily consumption of calcium and iron than children who skipped breakfast (SMD, −12.650; 95%CI: −14.616, −10.685 and SMD, −19.534; 95%CI: −27.887, −11.181, respectively), the heterogeneity between studies being very high (*I*^2^ = 99.26; *p* < 0.001 and *I*^2^ = 99.91; *p* < 0.001, respectively). Likewise, as it is shown in Figure 3C,E, RTEC consumers had a significantly higher magnesium and potassium intake than breakfast skippers (SMD, −10.903; 95%CI: −18.078, −3.729) and (SMD, −6.972; 95%CI: −10.689, −3.254), respectively. The heterogeneity between studies was high (*I*^2^ = 99.96; *p* < 0.001 and *I*^2^ = 99.91; *p* < 0.001, respectively). Concerning sodium, no significant associations were observed between those children who usually consume RTEC breakfast and breakfast skippers (Figure 3D).

**Figure 4:**
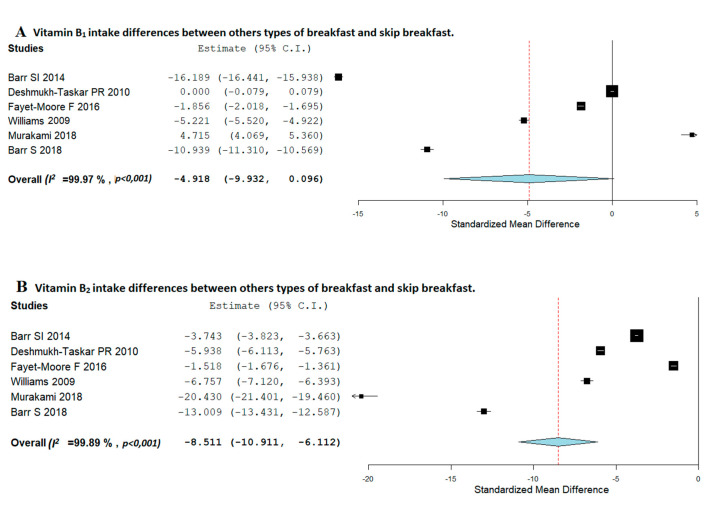

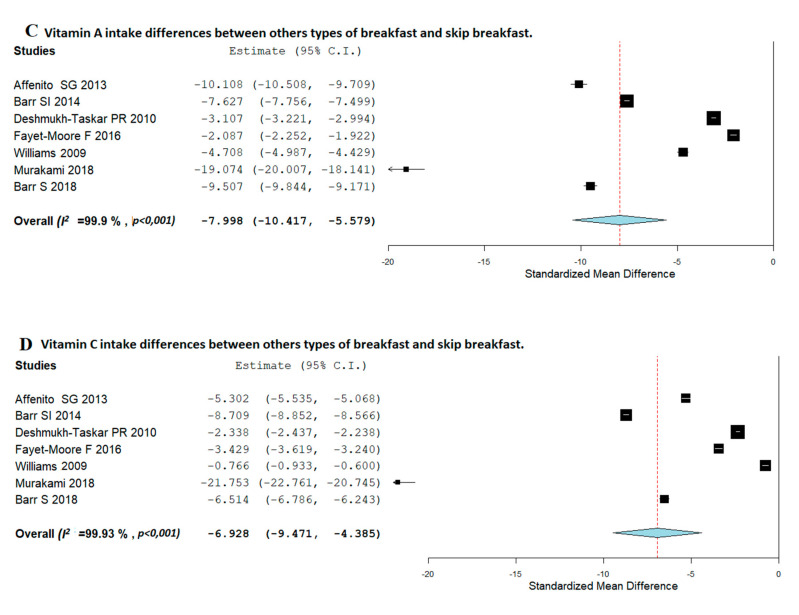
Random-effects meta-analysis of the effects of relationships between consumption of other types of breakfast and breakfast-skipping concerning vitamin B1 (**A**), vitamin B2 (**B**), vitamin A (**C**), and vitamin C (**D**) intake.

**Figure 5:**
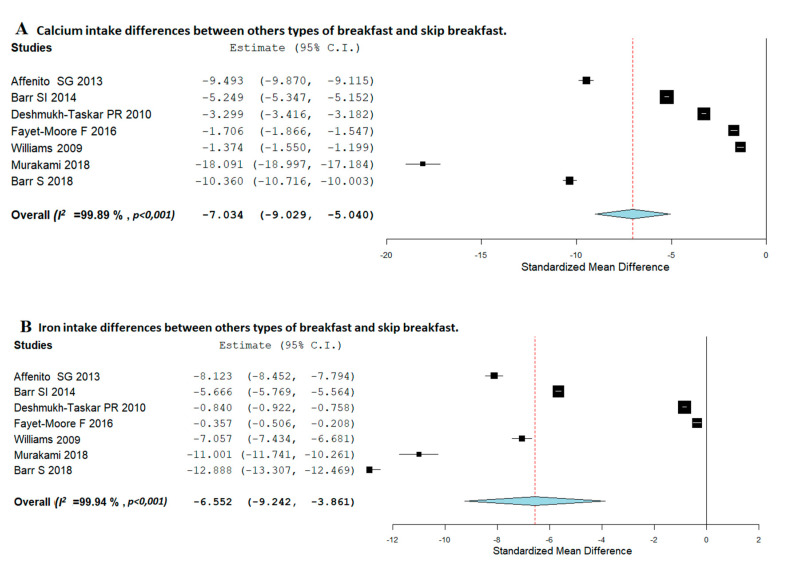

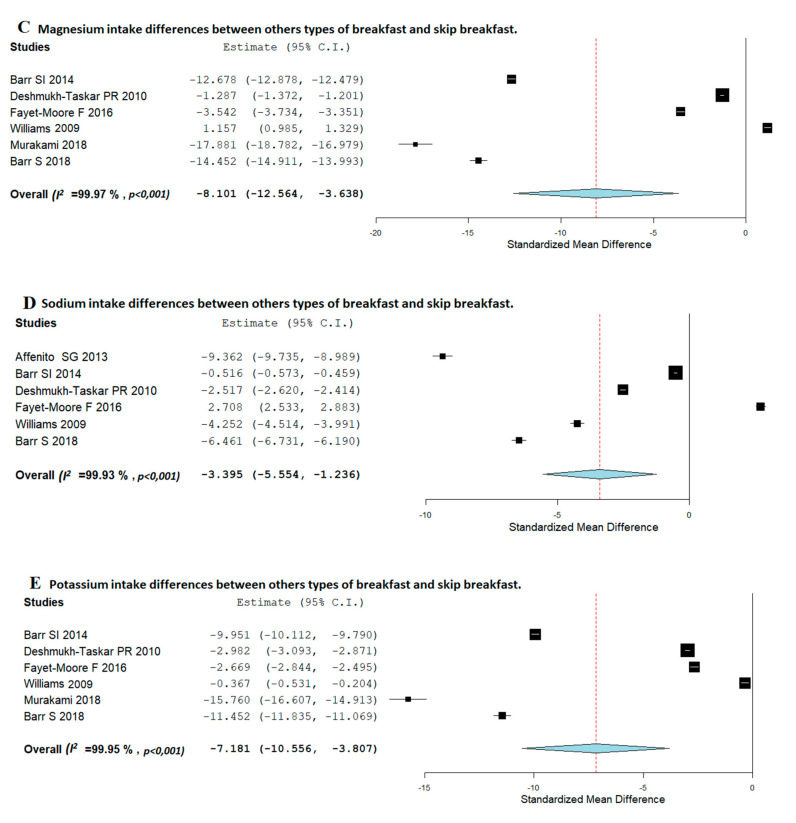
Random-effects meta-analysis of the effects of relationships between consumption of other types of breakfast and breakfast-skipping concerning calcium (**A**), iron (**B**), magnesium (**C**), sodium (**D**), and potassium (**E**) intake.

Differences in vitamins and minerals intake between consumption of other types of breakfast and skipping breakfast are shown in Figure 4 (vitamins) and Figure 5 (minerals).

Regarding thiamine (vitamin B1), no significant associations were observed between breakfast consumers and those children skipping breakfast (Figure 4A). Nevertheless, those children who eat breakfast had a higher significantly daily riboflavin (vitamin B2) consumption than those children who usually skip breakfast (SMD, −8.511; 95%CI: −10.911, −6.112) (Figure 4B). The heterogeneity between studies was high (*I*^2^ = 99.89%; *p* < 0.001).

Regarding Vitamin A and C, Figure 4C,D shows that breakfast consumers had a significantly higher consumption of vitamin A and C than those children who usually skip breakfast (SMD, −7.998; 95%CI: −10.417, −5.579 and SMD, −6.928; 95%CI: −9.471, −4.385, respectively). The heterogeneity amongst studies was high, (*I*^2^ = 99.9%; *p* < 0.001) and (*I*^2^ = 99.93%; *p* < 0.001), respectively.

When analyzing minerals intake, Figure 5A,B shows that those who usually consume breakfast had significantly higher daily intake of calcium and iron than breakfast skippers, (SMD, −7.034; 95%CI: −9.029, −5.040) and (SMD, −6.552; 95%CI: −9.242, −3.861), respectively. The heterogeneity between studies was high, (*I*^2^ = 99.89%; *p* < 0.001) and (*I*^2^ = 99.94%; *p* < 0.001), respectively. Regarding magnesium intake (Figure 5C), breakfast consumers had a higher consumption in respect to breakfast skippers (SMD, −8.101; 95%CI: −12.564, −3.638). Nevertheless, the heterogeneity between studies was high (*I*^2^ = 99.97%; *p* < 0.001).

Likewise, as shown in Figure 5D,E, it was observed that breakfast consumers had higher consumption of sodium and potassium in respect to breakfast skippers, (SMD, −3.395; 95%CI: −5.554, −1.236) and (SMD, −7.181; 95%CI: −10.556, −3.807), respectively. The heterogeneity amongst studies was high, (*I*^2^ = 99.93%; *p* < 0.001) and (*I*^2^ = 99.95%; *p* < 0.001), respectively.

## 4. Discussion

To the author’s knowledge, this is the first SR taking into consideration the association between breakfast consumption and daily micronutrients intake. The most important finding was the association between breakfast consumption and a higher daily vitamins and minerals intake in both children and adolescents.

Articles included assessed diet using individual or combined FR, 24h-DR, and FFQ. The most common strategy to collect dietary information was through face-to-face interview (*n* = 11; 33.33%). Nine studies (27.27%) included a self-reported questionnaire, five studies included caregiver-reported questionnaires (15.15%), and eight studies (24.24%) a combination of self- and caregiver-reported questionnaires. Likewise, when assessing dietary intake, it is important to evaluate how nutrients were computed; however, only a few studies (*n* = 7) reported the reference of the used food composition tables [28,50,62,63,65,67,68].

Most of the studies included in this SR compared the dietary intake of breakfast consumers or consumers of different types of breakfast, for instance RTEC-based breakfast with breakfast-skippers. Actually, of the 30 included studies, the main issue was breakfast’s RTEC consumption (*n* = 22; 66.66%). RTEC include many types of cereals, refined, whole grain, sweetened, and unsweetened, but in the markets and industry predominantly exists RTEC refined and sweetened. Several previous studies recognized that RTEC consumption at breakfast is associated with a healthier diet [32,52,54,56,67,71] but, in a previous review, authors suggested that RTEC with functional and best nutrient profiles should be developed [72]. In another review, RTEC consumption was related with a healthier dietary pattern (DP), nevertheless, total sugar intake was also high [73]. Some RTEC have high fiber content and have been fortified with essential nutrients as vitamins and minerals, but usually they are low in fat and high in carbohydrates, polysaccharides and sugar [59,74]. Previous studies suggested that RTEC could promote breakfast consumption as RTEC consumption was frequent among breakfast consumers [75]. Nevertheless, for future studies it would be important to investigate or differentiate the cereal consumed e.g., oats, muesli or RTEC sweetened, as not all types of cereals are good sources of micro- and macro-nutrients [46].

### 4.1. Breakfast Consumption and Vitamins Intake

Taking into consideration some specific foods consumed at breakfast, such as RTEC, some studies compared children or adolescents’ RTECs with non-RTEC consumers at breakfast. Some authors showed associations between RTEC consumption and vitamins intake. Five articles showed that RTEC consumers, both in children and adolescents [44,50,53,56,58], had a higher vitamin A intake than those who usually skip breakfast. Papoutsou et al. showed that those who eat milk and pastries or other types of breakfast had lower intakes of vitamin A [66] than those children who eat regularly RTEC at breakfast. Regarding B vitamins intake, Morgan et al. [49] observed that children who eat RTEC three or more times per week had a higher intake of the B vitamins group. Also, children and adolescents who usually consume RTEC had a high intake of the following B-vitamins: thiamine [42,50,52,54,56,60,61,62,67], riboflavin [42,44,48,50,52,54,56,58,60,61,62,67], niacin [42,44,56,58,60,67], pyridoxine [42,44,50,54,56,60,62], biotin [48], folate [29,42,44,50,52,53,56,59,61,62,64], and cobalamin [44,52,53,54,58,64]. Furthermore, our meta-analysis showed similar results for the difference in micronutrients intake between children consuming RTEC breakfast and those skipping breakfast. In children, those who usually consume RTEC had a high intake of thiamine, riboflavin, vitamin A, and vitamin C.

In the same way, in both children and adolescents, RTEC consumers compared with those who consume other types of breakfast had higher intakes of thiamine, riboflavin, pyridoxine, folate, vitamin A, and vitamin C [42,66]. These results are in line with the frequent fortification of RTEC with vitamins and other micronutrients [59,74]. Previous research showed that the fortified cereal’s consumption with milk produces some benefits in terms of the vitamins and iron intake, and significant improvement in biomarkers of nutritional status [76].

Different authors observed significant associations between breakfast consumption and vitamins intake. Breakfast consumers had a higher intake of vitamin A [41,43,51,68], thiamine [16,43,46,51,68], riboflavin [16,43,46,51], niacin [16,46,51], folate [16,46,51,68], and vitamin D [16,43] than breakfast skippers. Furthermore, an additional included study observed that regular breakfast consumption is associated with higher intakes and higher blood concentrations of some vitamins [16]. Additionally, our meta-analysis presented similar results. Breakfast consumers had a higher intake of riboflavin, vitamin A and vitamin C.

Breakfast consumption has been consistently associated with a better favorable nutrient intake [16,42]; in this sense, a previous study observed that breakfast skippers were significantly less likely to eat the recommended five servings of fruits and vegetables a day [16,77]. Furthermore, those children who usually consume breakfast usually consume more healthful breakfast foods like fruits, cereals, bread or milk [77].

### 4.2. Breakfast Consumption and Minerals Intake

Thirty-two articles (96.96%) investigated the association between breakfast consumption and minerals intake. Considering specific food groups consumed at breakfast, some authors observed a positive association between RTEC consumed at breakfast and the following minerals: calcium [42,44,48,50,54,56,58,59,60,67], iron [29,42,44,46,47,49,50,52,54,56,58,59,60,61,67], sodium [46], magnesium [42,50,54,56], zinc [50,54,56,59], potassium [42,46,48,54,58], phosphorous [42,46,48,50], iodine [46,62], and copper [50]. Also, two additional studies showed that calcium intake increased with cereal consumption [53,64]. Milk and dairy products used to be the major breakfast constituents in most of the countries [78,79] and provide a high source of vitamins and minerals such as calcium, vitamin D, magnesium, or phosphorous [80,81,82]. Furthermore, two articles showed that those who usually consume RTEC had a high consumption of milk and dairy products [48,57]. In contrast, Ortega et al. presented that dairy breakfast consumption was associated with high daily calcium intake, but this does not depend solely on breakfast consumption [63].

Children and adolescents who usually consume breakfast had higher intakes of calcium [3,30,41,43,45,46,68], iron [3,41,43,45,68], potassium [43,68], magnesium [43,68], zinc [43,68], and iodine [3] than breakfast skippers. In agreement with our SR, our meta-analysis presents similar results, breakfast consumers had a higher consumption of iron, calcium and magnesium. In the same way, Mattys et al. showed that adolescents with good quality breakfast consumed a higher intake of iron, magnesium and phosphorous [65].

It is important to mention that in a previous intervention study, [24] authors observed that adolescents who used to consume breakfast had more adequate vitamins and minerals intakes than those used to skip breakfast. It is worth noticing that approximately 92% of all RTEC are fortified with essential nutrients, however, in different previous studies, authors showed that children and adults who regularly consume RTEC increase their daily vitamins and minerals intake [31,83].

### 4.3. Potential Influencing Factors of Heterogeneity

We have to acknowledge that important heterogeneity was found for both types of analyzed groups, skipping breakfast versus RTEC breakfast or consumption of others types of breakfast. The high heterogeneity can be explained by the large range of subject’s age (2–16 years) and sample size of the included studies (min. 200, max. 12,281 participants) and the used dietary assessment method (24 h-DR, FR or FFQ). Responses may vary depending on the respondent of these questionnaires (children, adolescents, parents or caregivers) and the socio-cultural desirability. To address these differences, the included meta-analysis was done by age subgroups, showing no differences in their heterogeneity.

### 4.4. Strengths and Limitations

Our study has some limitations. Firstly, the studies’ design, as most of the included articles were cross-sectional, and it is not possible to determine cause–effect associations between exposure and outcome variables. Half of the studies were not performed in representative samples of the correspondent population, compromising their representativeness. Furthermore, the methodology has its own limitations, for example, some of the included articles evaluated the dietary intake with one 24 h-DR, which is not representative of the habitual diet. Nevertheless, this method is accepted for studying the intake in a large sample of the population and estimating the mean nutrients intake. Reported dietary intake could provide biased results (under or over-reporting) due to social desirability. Furthermore, we only included publications in English and Spanish, and we had to refuse two studies wrote in other different languages (Chinese and Korean). On the other hand, the number of comparison groups is large, making it difficult to obtain conclusions. Only one study analyzed the association between breakfast consumption and blood vitamins concentrations, and it was no possible to compare the results [16].

One of the strengths of the current manuscript is that, to our knowledge, this is the first systematic review and meta-analysis analyzing the associations between breakfast consumption and overall daily micronutrients intake. Also, the included studies were developed in four different continents (America, Asia, Europe and Oceania), which is interesting because they offer a global perspective. On the other hand, the population included in the studies were children and adolescents, and it is important because this means breakfast consumption during youth. Finally, some of the studies were included in the meta-analysis allowing to weight the effect of breakfast on micronutrient intake in young population.

## 5. Conclusions

Breakfast consumption seems to be associated with higher vitamins and minerals intake. On the other hand, the most frequent food consumed at breakfast was RTEC, and RTEC consumption at breakfast might have beneficial effects in daily vitamins and minerals intake. However, it is important to know the nutritional profile of the RTEC consumed, as they usually have a high-added sugars and/or fat content, and these components should be avoided.

Dietary habits change over the years, and therefore breakfast foods tend also to change. In this sense, it is essential to develop new studies to recognize breakfast consumption impact and its relationship with overall health. Additional studies should be developed to investigate the relationship of the different foods and beverages consumed at breakfast in terms of vitamins and minerals intake and their status.

## Figures and Tables

**Table 1:** Inclusion and exclusion criteria based on PICOS (Participants, Intervention, Control, Outcomes) principles.

PICOS	Inclusion Criteria	Exclusion Criteria
Participant	Population older than 2 years and younger than 18 years; both sexes; all nationalities	Population with different ages. Participants with any reported or known illness.
Intervention	Breakfast consumers (Ready to Eat Cereals (RTEC), other types of breakfast)	Not having breakfast data
Control/Comparator group	Breakfast skippers	Not having breakfast data
Outcome	Total daily intake of micronutrients.	Other outcomes not related with breakfast consumption

**Table 2:** Appraisal Tool for Cross-Sectional Studies (AXIS).

Assessment Criteria	No. of Satisfactory Studies
1. Were the aims/objectives of the study clear?	33
2. Was the study design appropriate for the stated aim(s)?	33
3. Was the sample size justified?	22
4. Was the target/reference population clearly defined? (Is it clear who the research was about?)	33
5. Was the sample frame taken from an appropriate population base so that it closely represented the target/reference population under investigation?	27
6. Was the selection process likely to select subjects/participants that were representative of the target/reference population under investigation?	24
7. Were the measures undertaken to address and categorize non-responders?	1
8. Were the risk factor and outcome variables measured appropriate to the aims of the study?	32
9. Were the risk factor and outcome variables measured correctly using instruments/measurements that had been trialed, piloted or published previously?	13
10. Is it clear what was used to determine statistical significance and/or precision estimates? (e.g., *p* values, Cis)	33
11. Were the methods (including statistical methods) sufficiently described to enable them to be repeated?	33
12. Were the basic data adequately described?	25
13. Does the response rate raise concerns about non-response bias?	1
14. If appropriate, was information about non-responders described?	0
15. Were the results internally consistent?	33
16. Were the results for the analyses described in the methods presented?	32
17. Were the authors’ discussions and conclusions justified by the results?	33
18. Were the limitations of the study discussed?	23
19. Were there any funding sources or conflicts of interest that may affect the authors’ interpretation of the results?	22
20. Was ethical approval or consent of participants attained?	28

**Table 3:** Characteristics, description and summary of outcomes of articles included in the systematic review (SR) on breakfast and micronutrients intake.

Author	Country, Year and Type of Study	Aim	Sample and Characteristics of Participants ^£^	Data Source and Dietary Assessment of Breakfast (BF)	Principal Outcome about Micronutrients
Affenito, S. et al. 2005 [30]	United States (US) N.A Longitudinal	To examine the association between BF frequency and Ca and fiber intake.	*n* = 2379 Girls. 9–19 y	National Heart, Lung, and Blood Institute Growth and Health Study 3 day-Food records	Frequent BF consumption was associated with more intake of Ca regardless of the total amount of EI consumed (*p* < 0.001).
Affenito S. et al. 2013 [41]	US 2004–2005 Cross-sectional	To examine the association of RTEC consumption and dietary nutrients intake.	*n* = 2298 5–18 y	The third School Nutrition Dietary Assessment Study 1–24 h dietary recall	BF consumption improves the intake of vitA, Ca and Fe (*p* < 0.05).
Afeiche, M. et al. 2017 [28]	Mexico, 2012 Cross-sectional	To compare BF dietary patterns (DP) with BF skipping and the associations with total-day diet energy and nutrients intake.	*n* = 3760 4–13 y	Mexican National Health and Nutrition Survey 1–24 h dietary recall	BF skippers consumed less B vitamins (riboflavin, niacin, pyridoxine, folate and cobalamin), Ca, vitD, Fe, Zn, Na, and K than BF consumers. The sweetened beverages and milk and sweetened breads DP had the lowest intakes of Fe, Zn and K at BF.
Albertson A. et al. 2003 [56]	US 1998–1999 Cross-sectional	To assess the relationship between RTEC frequency consumption and nutrients intake in children.	*n* = 603 4–12 y	American household surveys 14–Food records	RTEC frequency of consumption was not associated to Na, vitE, but it was associated with high intake of vitA, pyridoxine, vitC, thiamin, riboflavin, niacin, folate, Ca, Mg, Fe and Zn (*p* = 001).
Albertson A. et al. 2008 [57]	US 1987 Longitudinal	To assess the association between RTEC consumption and energy and nutrients intake.	*n* = 2379 9–10 y	The National Heart, Lung, and Blood Institute Growth Health Study 1–24 h dietary recalls	RTEC consumers did not differ from non-RTEC consumers in Na intake (N.S).
Balvin Frantzen, L. et al. 2013 [58]	US 2001–2004 Longitudinal	To assess the association between frequency of RTEC consumption and nutrients intake.	*n* = 625 Mean age = 9.13 y	BIENESTAR Study 3–24 h dietary recalls	Baseline data analysis concludes that frequency of RTEC consumption was positively associated with the intake of vitD, vitC, riboflavin, niacine, cobalamin, Ca, Fe, and K. (*p* < 0.05).
Barr, S. et al. 2014 [42]	Canada 2004 Cross-sectional	To assess the effect of skipping BF, consuming BF and consuming BF with RTEC on intake of nutrients.	*n* = 12,281 4–18 y	Canadian Community Health Survey, 2004. 1–24 h dietary recall	RTEC consumers had a higher intake of thiamin, pyridoxine, vitD, Ca, Fe, Mg, P, and K than other-BF consumers and non-BF consumers. Both BF consumers and RTEC consumers had higher intakes of vitA, folate and vitC compared with BF skippers. RTEC consumers had higher intakes of riboflavin than non-RTEC consumers and BF skippers. Other-BF consumers had higher intake of niacin than other groups (*p* < 0.05).
Barr, SI. et al 2018 [43]	Canada 2015 Cross-sectional	To compare daily EI and nutrients intake of BF consumers and BF skippers.	*n* = 2331 6–12 y *n* = 2026 13–17 y	Canadian Community Health Survey-Nutrition 1–24 h dietary recall	Children and adolescents who usually consume BF had a higher intake of thiamin, vitC, Fe, and Mg (*p* < 0.5). In adolescents, BF consumers had a high intake of vitA, riboflavin, cobalamin, vitD and K (*p* < 0.01), pyridoxine, Ca (*p* < 0.01), and Zn (*p* < 0.5).
Barton, B. et al. 2005 [59]	US 1985 Cross-sectional	To assess the association of BF and RTEC consumption with intake of nutrients.	*n* = 2379 9–19 y	National Heart, Lung and Blood Institute Growth and Health study 3–24 h dietary recalls	RTEC consumption increases the intake of Ca, Fe, folate, vitC, and Zn.
Coulthard, J. et al. 2017 [3]	United Kingdom (UK) 2008–2012 Cross-sectional	To assess differences in nutrients intake between BF skippers and BF consumers.	*n* = 1686 4–18 y	National Diet and Nutrition Survey 4–Food records	Children and adolescents had significantly higher mean intakes of folate, Ca and I, and significantly lower mean intakes of Na for days on which BF was consumed compared with non-BF days. Frequency of BF consumption was positively associated with folate, Na, Ca, Fe, and I (*p* < 0.05).
Deshmukh-Taskar, P. et al. 2010 [70]	US 1999–2006 Cross-sectional	To assess the relationship between skipping BF or having lunch with nutrients intake, nutrient adequacy and adiposity.	*n* = 930 9–13 y *n* = 1805 14–18 y	NHANES ^Ω^ 1–24 h dietary recall	In both children and adolescents, BF skippers had a lower intake of vitA, vitC, riboflavin, cobalamin, folate, Ca, P, Mg, K, and Zn than the other groups (*p* < 0.05). RTEC consumers had lower Na intake than the other groups (*p* < 0.05). Thiamine, niacin, pyridoxine and Fe intake was significantly higher in the RTEC consumers group (*p* < 0.05).
Fayet-Moore, F. et al. 2016 [46]	Australia 2007 Cross-sectional	To assess the impact of BF skipping, BF with RTEC and BF without RTEC on nutrient intake.	*n* = 4487 2–16 y	Australian National Children’s Nutrition and Physical Activity Survey 2–24 h dietary recall	BF consumers had a higher intake of total Ca, folate, Mg and Zn than BF skippers. RTEC consumers had higher intake of Fe, P, K, I, and Na (*p* < 0.001). BF consumers, and to a higher degree RTEC consumers, were more likely to meet the EAR of Ca and Fe than BF skippers (*p* < 0.001).
Fayet-Moore, F. et al. 2017 [45]	Australia 2011–2012 Cross-sectional	To investigate the impact of BF skipping, BF with RTEC and BF without RTEC on daily nutrients intake.	*n* = 2821 2–18 y	National Nutrition and Physical Activity Survey 1–24 h dietary recall	BF skippers had lower niacin, Fe, thiamin, riboflavin, folate and Ca, intake whereas they had a higher Na intake than BF consumers (*p* < 0.001).
Fulgoni, VL. et al. 2019 [55]	US 2011–2014 Cross sectional	To compare diet quality and nutrient intake among children consuming an oatmeal-containing BF versus those of children consuming other popular BF.	*n* = 5876 2–18 y	NHANES ^Ω^ 1–24 h dietary recall	Oatmeal consumers had a significantly higher intake of Ca, Fe, Mg, K, folate, vitA, and vitD than BF skippers. In children and adolescents, RTEC consumers had a higher intake of Ca, Fe, Mg, K, vitA, and vitE than those who usually consume “Doughnuts, sweets rolls and pastries”; higher intake of Mg and K than those who usually consume “Pancakes, waffles, French toast” and higher intakes of Ca, Fe and Mg than consumers of “Eggs and omelettes”. RTEC consumers (lower and higher sugar) had a higher intake of Fe and folate than those children and adolescents who usually consume oatmeal BF.
Gibson, S. et al. 1995 [60]	UK N.A Cross-sectional	To examine the relation ship between RTEC frequency of consumption and total daily nutrients intake.	*n* = 2705 10–15 y	7-day weighed records	Frequency of RTEC consumption in boys and girls was positively associated with Ca, Fe, thiamin, riboflavin, niacin, and pyridoxine intake.
Gibson, S. et al. 1999 [47]	UK N.A Cross-sectional	To examine associations between RTEC consumption and iron intake.	*n* = 904 1.5–4.5 y	UK National Diet and Nutrition Survey 4 day weighed records	High RTEC consumers had significantly higher Fe intakes than low cereal consumers. (*p* < 0.0001). They did not have a significantly higher intake of Ca compared to the other groups (*p* = 0.06).
Gibson, S. et al. 2003 [61]	UK N.A Cross-sectional	To examine the impact of RTEC on micronutrient status.	*n* = 1688 4–18 y	The National Diet and Nutrition Survey of Young People 7–24 h dietary recalls	The highest tertile of RTEC intake had 20–60% higher intake of iron, B vitamins and vitD, compared with the first tertile. A positive association between Fe, thiamin, riboflavin and folate was observed across tertiles of RTEC consumption (*p* < 0.001).
Matthys, C. et al. 2007 [65]	Belgium 1997 Cross-sectional	To describe BF consumption patterns and overall nutrients profile.	*n* = 341 13–18 y	Food Consumption Survey 7–24 h dietary recalls	Good quality of BF was associated with higher intake of Ca, Mg, thiamin, riboflavin and vitC and P compared to low quality of BF consumers (*p* < 0.001). Specifically, girls with good quality of BF had a significantly higher intake of Ca, P, Fe, Mg, thiamin, riboflavin, and vitC (*p* < 0.001).
McNulty, H. et al. 1996 [64]	Ireland 1990 Cross-sectional	To establish the contribution of RTEC to the overall nutrients intake.	*n* = 1015 12–15 y	1–24 h dietary recall	Higher RTEC consumption was associated with higher cobalamin intake, except for boys aged 12 years. Folate intake significantly increased with increasing intake of fortified BF cereals in the younger adolescents (*p* < 0.05). Ca intake increased with increasing BF cereals intake in all age and sex groups (*p* < 0.05).
Michels, N. et al. 2015 [48]	Europe 2006–2007 Cross-sectional	To analyze the association of RTEC consumption frequency with dietary intake.	*n* = 1215 12.5–17.5 y	HELENA Study ^Ω^ 1 Food Frequency Questionnaire 2–24 h dietary recalls	Ca, P, K, riboflavin, pantothenic acid, biotin, and VitD intakes were significantly higher in the RTEC consumers group (*p* < 0.05).
Mielgo-Ayuso, J. et al. 2017 [16]	European countries 2006–2007 Cross-sectional	To examine the association between BF consumption patterns and vitamins intake	*n* = 1058 12.5–17.5 y	HELENA Study ^Ω^ 2–24 h dietary recalls	BF consumption was associated with high intakes of vitD and folate in both sexes, with high intakes of pyridoxine and vitE in girls (*p* < 0.05).
Mohd Nasir, M.T. et al. 2017 [67]	Malaysia 2013 Cross-sectional	To compare foods consumed at breakfast and nutrient intake for the total day between RTEC consumers and non-RTEC consumers	*n* = 1819 6–12 y	MyBreakfast study Children 6–9 y: 2 day food records Children 10–12 y: 2–24 h dietary recalls	RTEC consumers had a higher daily intake of vitC, thiamine, riboflavin, niacin, Ca, and Fe (*p* < 0.05). Non-RTEC consumers had a higher intake of Na than RTEC consumers (*p* < 0.05). No significant associations in vitA and P were observed between RTEC consumers and non-RTEC consumers.
Morgan, K.J. et al. 1981 [49]	US 1977 Cross-sectional	To assess BF consumption pattern and relate it with nutrients intake	*n* = 657 5–12 y	7 day food records	BF had a significant contribution to child’s daily nutrients intake. RTEC consumers for 3 or more times per week had higher intake of Fe and B vitamins (*p* < 0.001) than non-RTEC consumers.
Murakami, K. et al. 2018 [68]	Japan 2012 Cross-sectional	To assess BF consumption and its association with daily dietary intake of nutrients, food groups and overall diet quality.	*n* = 1444 6–11 y *n* = 1134 12–17 y	National Health and Nutrition Survey 2012 1 Dietary record	BF consumers had higher intakes of vitK, folate, vitC, Ca, Mg, and P in both age groups. Children who usually consumed BF had a higher intake of vitA, vitK and vitC than BF skippers (*p* < 0.05). In adolescents, BF consumers had a higher intake of K and Fe than BF skippers (*p* < 0.05). In both children and adolescents, BF skippers had a lower intake of pyridoxine, folate, pantothenic acid, Ca, Mg, P, and Zn than BF consumers (*p* < 0.05).
Ortega, RM. Et al. 1996 [62]	Spain N.A Cross-sectional	To analyze the influence of RTEC consumption at BF upon dietary habits.	*n* = 200 9–13 y	4–24 h dietary recalls	The intake of thiamine, pyridoxine, folate, β-carotene (*p* < 0.05), riboflavin, and I (*p* < 0.1) was higher in the group of RTEC consumers.
Ortega, RM. et al. 1998 [63]	Spain N.A Cross-sectional	To assess the association between Ca and milk products consumed at BF with their total daily intake.	*n* = 200 9–13 y	7–24 h dietary recalls	BF with <20% of total EI included lower quantities of Ca than larger BF (*p* < 0.05). These adolescents also consumed less Ca over the rest of the day.
Papoutsou, S. et al. 2014 [66]	Cyprus 2007–2008 Cross-sectional	To investigate the association of BF pattern consumption with children’s diet quality in a sample from Cyprus.	*n* = 1558 4–8 y	IDEFICS Study ^Ω^ 1–24 h dietary recall	Milk and pastry consumers had lower intake of Fe, Na, thiamin, riboflavin, and pyridoxine than RTEC consumers. Other—BF, milk and pastry consumers had lower intakes of vitA and vitC. Milk consumers had a lower intake of Mg than the other groups. Milk and pastry consumers had a higher intake of Mg and P (*p* < 0.05).
Preziosi, P. et al. 1999 [50]	France N.A Cross-sectional	To examine the associations between the intake of different types of BF and dietary intakes.	*n* = 1108 2–18 y	1–24 h dietary recall	Percent of RDA for Ca, P, magnesium, and Fe were exceeded in non-RTEC and RTEC consumers but was significantly higher in the group of RTEC consumers (*p* < 0.05). RDA for thiamine and riboflavin were also reached in both groups but RTEC consumers had higher intakes of both nutrients (*p* < 0.001 and *p* < 0.01). Percent of RDA for magnesium, Zn, copper, pyridoxine, folate, vitC, vitA, and vitE were also higher in the RTEC group.
Ramsay, SA. Et al. 2018 [51]	US 2005–2012 Cross-sectional	To examine food intake, nutrients intake and overall diet quality among BF consumers and BF skippers.	*n* = 8590 2–12 y	NHANES ^Ω^ 1–24 h dietary recall	BF skippers did not meet the average amount of nutrients of children who consumed BF. BF skippers consumed less vitA, folate, Fe, and Ca than those who consumed BF.
Ruxton, CH. et al. 1996 [29]	UK Scotland 1991–1992 Cross-sectional	To provide new data on the BF habit of children	*n* = 136 5–9 y	7–24 h dietary recall	The overall diets of children in the high RTEC group were higher in micronutrients than the other groups. Folate and Fe in the low RTEC group were below recommendations.
Vatanparast, H. et al. 2019 [54]	Canada 2015 Cross-sectional	To evaluate how RTEC consumption contributed to daily energy and nutrient intakes and then compare them with non-consumers	*n* = 3810 6–12 y *n* = 2379 12–18 y	The Canadian diet, the recent nationally representative dietary survey 1–24 h dietary recall	Children and adolescents who usually consume RTEC had significantly higher intake of pyridoxine, vitD, riboflavin, thiamine, K, Ca, Fe, and Mg than non-RTEC consumers. In children, RTEC consumers had a higher intake of cobalamin and Zn than non-RTEC consumers. In adolescents, RTEC consumers had a lower intake of Na than non-RTEC consumers.
Williams, B.M. et al. 2009 [52]	US 1999–2002 Cross-sectional	To assess if BF dietary patterns are associated with nutrients intake and nutritional adequacy.	*n* = 1389 2–12 y	NHANES ^Ω^ 1–24 h dietary recall	RTEC consumers had a higher intake of vitA, cobalamin, thiamine, riboflavin, folate, and Fe (*p* ≤ 0.05).
Williams, P. et al. 2007 [53]	Australia 1995 Cross-sectional	To assess the contribution of BF to the nutrition of Australian children and adolescents.	*n* = 3007 2–18 y	National Health Survey 1–24 h dietary recall 1 FFQ	Higher RTEC consumption was associated with higher cobalamin intake, except for boys aged 12 years. Folate intake significantly increased with increasing intake of RTEC in younger adolescents. (*p* < 0.05). Ca intake increased with increasing BF cereal intake in all age and sex groups. (*p* < 0.05).

£ = All the studies included boys and girls in their analysis, except those in which it was specified that only one gender was included. Abbreviations: N.A: Not available; BF: Breakfast; Y = Years; EI: Energy intake; RTEC: Ready to eat cereal; DP: Dietary Pattern; RDA: recommended dietary allowances; vitA: vitamin A; vitC: vitamin C; vitE: vitamin E; vitD: vitamin D; vitK: vitamin K; Mg: magnesium; Zn: zinc; Ca: Calcium; Na: Sodium; K: potassium; Fe: iron; I: Iodine; P: Phosphorus; Ω = IDEFICS—Identification and prevention of dietary- and lifestyle-induced health effects in children and infants; HELENA—Healthy Lifestyle in Europe by Nutrition in Adolescence.; NHANES—National Health and Nutrition Examination Survey; FFQ: Food Frequency Questionnaire.

## Supplementary Materials

The following are available online at https://www.mdpi.com/2072-6643/12/10/3201/s1. Table S1. Quality appraisal findings.

## Abbreviations

95%CI	95% confidence intervals
24 h-DR	24 h-dietary recalls
μg	Microgram
AXIS	The Appraisal tool for Cross-sectional Studies
BF	Breakfast
DP	Dietary Pattern
EI	Energy Intake
FFQ	Food Frequency Questionnaire
FR	Food Records
IBRI	International Breakfast Research Initiative
MD	Mean Difference
mg	Milligrams
MUFA	Monounsaturated Fatty Acids
N.A	Not Available
PICO	Participants, Intervention, Control, Outcomes
PRISMA	Preferred Reporting Items For Systematic Reviews and Meta-Analysis.
PUFA	Polyunsaturated Fatty Acids
RTEC	Ready to eat cereal
SF	Saturated fat
SR	Systematic Review
UK	United Kingdom
US	United States
y	Years

## References

[1] O’neil C.E., Nicklas T.A., Fulgoni V.L. (2014). Nutrient Intake, Diet Quality, And Weight/Adiposity Parameters In Breakfast Patterns Compared With No Breakfast In Adults: National Health And Nutrition Examination Survey 2001–2008. J. Acad. Nutr. Diet..

[2] Rehm C.D., Drewnowski A. (2017). Replacing American Breakfast Foods With Ready-To-Eat (Rte) Cereals Increases Consumption Of Key Food Groups And Nutrients Among Us Children And Adults: Results Of An Nhanes Modeling Study. Nutrients.

[3] Coulthard J.D., Palla L., Pot G.K. (2017). Breakfast Consumption And Nutrient Intakes In 4-18-Year-Olds: Uk National Diet And Nutrition Survey Rolling Programme (2008–2012). Br. J. Nutr..

[4] O’neil C.E., Byrd-Bredbenner C., Hayes D., Jana L., Klinger S.E., Stephenson-Martin S. (2014). The Role Of Breakfast In Health: Definition And Criteria For A Quality Breakfast. J. Acad. Nutr. Diet..

[5] Szajewska H., Ruszczynski M. (2010). Systematic Review Demonstrating That Breakfast Consumption Influences Body Weight Outcomes In Children And Adolescents In Europe (Structured Abstract). Crit. Rev. Food Sci. Nutr..

[6] Gibney M.J., Barr S.I., Bellisle F., Drewnowski A., Fagt S., Hopkins S., Livingstone B., Varela-Moreiras G., Moreno L., Smith J. (2018). Towards An Evidence-Based Recommendation For A Balanced Breakfast-A Proposal From The International Breakfast Research Initiative. Nutrients.

[7] Monzani A., Ricotti R., Caputo M., Solito A., Archero F., Bellone S., Prodam F. (2019). A Systematic Review Of The Association Of Skipping Breakfast With Weight And Cardiometabolic Risk Factors In Children And Adolescents. What Should We Better Investigate In The Future?. Nutrients.

[8] Hallstrom L., Labayen I., Ruiz J.R., Patterson E., Vereecken C.A., Breidenassel C., Gottrand F., Huybrechts I., Manios Y., Mistura L. (2013). Breakfast Consumption And Cvd Risk Factors In European Adolescents: The Helena (Healthy Lifestyle In Europe By Nutrition In Adolescence) Study. Public Health Nutr..

[9] Gilardini L., Croci M., Pasqualinotto L., Caffetto K., Invitti C. (2015). Dietary Habits And Cardiometabolic Health In Obese Children. Obes. Facts.

[10] Karatzi K., Moschonis G., Choupi E., Manios Y. (2017). Late-Night Overeating Is Associated With Smaller Breakfast, Breakfast Skipping, And Obesity In Children: The Healthy Growth Study. Nutrition.

[11] Tin S.P., Ho S.Y., Mak K.H., Wan K.L., Lam T.H. (2011). Breakfast Skipping And Change In Body Mass Index In Young Children. Int. J. Obes..

[12] Mekary R.A., Giovannucci E., Cahill L., Willett W.C., Van Dam R.M., Hu F.B. (2013). Eating Patterns And Type 2 Diabetes Risk In Older Women: Breakfast Consumption And Eating Frequency. Am. J. Clin. Nutr..

[13] Mekary R.A., Giovannucci E., Willett W.C., Van Dam R.M., Hu F.B. (2012). Eating Patterns And Type 2 Diabetes Risk In Men: Breakfast Omission, Eating Frequency, And Snacking. Am. J. Clin. Nutr..

[14] Donin A.S., Nightingale C.M., Owen C.G., Rudnicka A.R., Perkin M.R., Jebb S.A., Stephen A.M., Sattar N., Cook D.G., Whincup P.H. (2014). Regular Breakfast Consumption And Type 2 Diabetes Risk Markers In 9- To 10-Year-Old Children In The Child Heart And Health Study In England (Chase): A Cross-Sectional Analysis. PLoS Med..

[15] Deshmukh-Taskar P., Nicklas T.A., Radcliffe J.D., O’neil C.E., Liu Y. (2013). The Relationship Of Breakfast Skipping And Type Of Breakfast Consumed With Overweight/Obesity, Abdominal Obesity, Other Cardiometabolic Risk Factors And The Metabolic Syndrome In Young Adults. The National Health And Nutrition Examination Survey (Nhanes): 1999–2006. Public Health Nutr..

[16] Mielgo-Ayuso J., Valtueña J., Cuenca-García M., Gottrand F., Breidenassel C., Ferrari M., Manios Y., De Henauw S., Widhalm K., Kafatos A. (2017). Regular Breakfast Consumption Is Associated With Higher Blood Vitamin Status In Adolescents: The Helena (Healthy Lifestyle In Europe By Nutrition In Adolescence) Study. Public Health Nutr..

[17] Shenkin A. (2006). Micronutrients In Health And Disease. Postgrad. Med. J..

[18] Lee H.A., Kim Y.J., Lee H., Gwak H.S., Park E.A., Cho S.J., Oh S.Y., Ha E.H., Kim H.S., Park H. (2013). Association Of Vitamin D Concentrations With Adiposity Indices Among Preadolescent Children In Korea. J. Pediatr. Endocrinol. Metab..

[19] Kaganov B., Caroli M., Mazur A., Singhal A., Vania A. (2015). Suboptimal Micronutrient Intake Among Children In Europe. Nutrients.

[20] Lee H.A., Park H. (2017). The Mediation Effect Of Individual Eating Behaviours On The Relationship Between Socioeconomic Status And Dietary Quality In Children: The Korean National Health And Nutrition Examination Survey. Eur. J. Nutr..

[21] Rampersaud G.C. (2009). Benefits Of Breakfast For Children And Adolescents: Update And Recommendations For Practitioners. Am. J. Lifestyle Med..

[22] Adolphus K., Lawton C.L., Champ C.L., Dye L. (2016). The Effects Of Breakfast And Breakfast Composition On Cognition In Children And Adolescents: A Systematic Review. Adv. Nutr..

[23] Vereecken C., Dupuy M., Rasmussen M., Kelly C., Nansel T.R., Al Sabbah H., Baldassari D., Jordan M.D., Maes L., Niclasen B.V. (2009). Breakfast Consumption And Its Socio-Demographic And Lifestyle Correlates In Schoolchildren In 41 Countries Participating In The Hbsc Study. Int. J. Public Health.

[24] Nicklas T.A., Reger C., Myers L., O’neil C. (2000). Breakfast Consumption With And Without Vitamin-Mineral Supplement Use Favorably Impacts Daily Nutrient Intake Of Ninth-Grade Students. J. Adolesc. Health.

[25] Faci M., Requejo A.M., Mena M.C., Navia B., Bermejo L.M., Perea J.M. (2001). Relationship Between The Caloric Content Of Breakfast And Dietary Habits Among School-Age Children. Rev. Esp. Nutr. Comunitaria.

[26] Monteagudo C., Palacin-Arce A., Mdel M.B., Pons A., Tur J.A., Olea-Serrano F., Mariscal-Arcas M. (2013). Proposal For A Breakfast Quality Index (Bqi) For Children And Adolescents. Public Health Nutr..

[27] Affenito S.G. (2007). Breakfast: A Missed Opportunity. J. Am. Diet. Assoc..

[28] Afeiche M.C., Taillie L.S., Hopkins S., Eldridge A.L., Popkin B.M. (2017). Breakfast Dietary Patterns Among Mexican Children Are Related To Total-Day Diet Quality. J. Nutr..

[29] Ruxton C.H., O’sullivan K.R., Kirk T.R., Belton N.R., Holmes M.A. (1996). The Contribution Of Breakfast To The Diets Of A Sample Of 136 Primary-Schoolchildren In Edinburgh. Br. J. Nutr..

[30] Affenito S.G., Thompson D.R., Barton B.A., Franko D.L., Daniels S.R., Obarzanek E., Schreiber G.B., Striegel-Moore R.H. (2005). Breakfast Consumption By African-American And White Adolescent Girls Correlates Positively With Calcium And Fiber Intake And Negatively With Body Mass Index. J. Am. Diet. Assoc..

[31] Rampersaud G.C., Pereira M.A., Girard B.L., Adams J., Metzl J.D. (2005). Breakfast Habits, Nutritional Status, Body Weight, And Academic Performance In Children And Adolescents. J. Am. Diet. Assoc..

[32] Michels N., De Henauw S., Beghin L., Cuenca-Garcia M., Gonzalez-Gross M., Hallstrom L., Kafatos A., Kersting M., Manios Y., Marcos A. (2016). Ready-To-Eat Cereals Improve Nutrient, Milk And Fruit Intake At Breakfast In European Adolescents. Eur. J. Nutr..

[33] Alexy U., Wicher M., Kersting M. (2010). Breakfast Trends In Children And Adolescents: Frequency And Quality. Public Health Nutr..

[34] Bellisle F., Rolland-Cachera M.F. (2007). Three Consecutive (1993, 1995, 1997) Surveys Of Food Intake, Nutritional Attitudes And Knowledge, And Lifestyle In 1000 French Children, Aged 9–11 Years. J. Hum. Nutr. Diet..

[35] Van Den Boom A., Serra-Majem L., Ribas L., Ngo J., Perez-Rodrigo C., Aranceta J., Fletcher R. (2006). The Contribution Of Ready-To-Eat Cereals To Daily Nutrient Intake And Breakfast Quality In A Mediterranean Setting. J. Am. Coll. Nutr..

[36] Giménez-Legarre N., Flores-Barrantes P., Miguel-Berges M.L., Moreno L.A., Santaliestra-Pasías A.M. (2020). Breakfast Characteristics And Their Association With Energy, Macronutrients, And Food Intake In Children And Adolescents: A Systematic Review and Meta-Analysis. Nutrients.

[37] Moher D., Shamseer L., Clarke M., Ghersi D., Liberati A., Petticrew M., Shekelle P., Stewart L.A., Group P.-P. (2015). Preferred Reporting Items For Systematic Review And Meta-Analysis Protocols (Prisma-P) 2015 Statement. Syst. Rev..

[38] Boudin F., Nie J.Y., Bartlett J.C., Grad R., Pluye P., Dawes M. (2010). Combining Classifiers For Robust Pico Element Detection. Bmc Med. Inform. Decis. Mak..

[39] Downes M.J., Brennan M.L., Williams H.C., Dean R.S. (2016). Development Of A Critical Appraisal Tool To Assess The Quality Of Cross-Sectional Studies (Axis). Bmj Open.

[40] Higgins J.P., Thompson S.G., Deeks J.J., Altman D.G. (2003). Measuring Inconsistency In Meta-Analyses. Bmj.

[41] Affenito S.G., Thompson D., Dorazio A., Albertson A.M., Loew A., Holschuh N.M. (2013). Ready-To-Eat Cereal Consumption And The School Breakfast Program: Relationship To Nutrient Intake And Weight. J. Sch. Health.

[42] Barr S.I., Difrancesco L., Fulgoni V.L. (2014). Breakfast Consumption Is Positively Associated With Nutrient Adequacy In Canadian Children And Adolescents. Br. J. Nutr..

[43] Barr S.I., Vatanparast H., Smith J. (2018). Breakfast In Canada: Prevalence Of Consumption, Contribution To Nutrient And Food Group Intakes, And Variability Across Tertiles Of Daily Diet Quality. A Study From The International Breakfast Research Initiative. Nutrients.

[44] Deshmukh-Taskar P.R., Nicklas T.A., O’neil C.E., Keast D.R., Radcliffe J.D., Cho S. (2010). The Relationship Of Breakfast Skipping And Type Of Breakfast Consumption With Nutrient Intake And Weight Status In Children And Adolescents: The National Health And Nutrition Examination Survey 1999–2006. J. Am. Diet. Assoc..

[45] Fayet-Moore F., Mcconnell A., Tuck K., Petocz P. (2017). Breakfast And Breakfast Cereal Choice And Its Impact On Nutrient And Sugar Intakes And Anthropometric Measures Among A Nationally Representative Sample Of Australian Children And Adolescents. Nutrients.

[46] Fayet-Moore F., Kim J., Sritharan N., Petocz P. (2016). Impact Of Breakfast Skipping And Breakfast Choice On The Nutrient Intake And Body Mass Index Of Australian Children. Nutrients.

[47] Gibson S.A. (1999). Iron Intake And Iron Status Of Preschool Children: Associations With Breakfast Cereals, Vitamin C And Meat. Public Health Nutr..

[48] Michels N., De Henauw S., Breidenassel C., Censi L., Cuenca-Garcia M., Gonzalez-Gross M., Gottrand F., Hallstrom L., Kafatos A., Kersting M. (2015). European Adolescent Ready-To-Eat-Cereal (Rtec) Consumers Have A Healthier Dietary Intake And Body Composition Compared With Non-Rtec Consumers. Eur. J. Nutr..

[49] Morgan K.J., Zabik M.E., Leveille G.A. (1981). The Role Of Breakfast In Nutrient Intake Of 5- To 12-Year-Old Children. Am. J. Clin. Nutr..

[50] Preziosi P., Galan P., Deheeger M., Yacoub N., Drewnowski A., Hercberg S. (1999). Breakfast Type, Daily Nutrient Intakes And Vitamin And Mineral Status Of French Children, Adolescents, And Adults. J. Am. Coll. Nutr..

[51] Ramsay S.A., Bloch T.D., Marriage B., Shriver L.H., Spees C.K., Taylor C.A. (2018). Skipping Breakfast Is Associated With Lower Diet Quality In Young Us Children. Eur. J. Clin. Nutr..

[52] Williams B.M., O’neil C.E., Keast D.R., Cho S., Nicklas T.A. (2009). Are Breakfast Consumption Patterns Associated With Weight Status And Nutrient Adequacy In African-American Children?. Public Health Nutr..

[53] Williams P. (2007). Breakfast And The Diets Of Australian Children And Adolescents: An Analysis Of Data From The 1995 National Nutrition Survey. Int. J. Food Sci. Nutr..

[54] Vatanparast H., Islam N., Patil R.P., Shamloo A., Keshavarz P., Smith J., Chu L.M., Whiting S. (2019). Consumption Of Ready-To-Eat Cereal In Canada And Its Contribution To Nutrient Intake And Nutrient Density Among Canadians. Nutrients.

[55] Fulgoni V.L., Brauchla M., Fleige L., Chu Y. (2019). Oatmeal-Containing Breakfast Is Associated With Better Diet Quality And Higher Intake Of Key Food Groups And Nutrients Compared To Other Breakfasts In Children. Nutrients.

[56] Albertson A.M., Anderson G.H., Crockett S.J., Goebel M.T. (2003). Ready-To-Eat Cereal Consumption: Its Relationship With Bmi And Nutrient Intake Of Children Aged 4 To 12 Years. J. Am. Diet. Assoc..

[57] Albertson A.M., Thompson D., Franko D.L., Kleinman R.E., Barton B.A., Crockett S.J. (2008). Consumption Of Breakfast Cereal Is Associated With Positive Health Outcomes: Evidence From The National Heart, Lung, And Blood Institute Growth And Health Study. Nutr. Res..

[58] Frantzen L.B., Trevino R.P., Echon R.M., Garcia-Dominic O., Dimarco N. (2013). Association Between Frequency Of Ready-To-Eat Cereal Consumption, Nutrient Intakes, And Body Mass Index In Fourth- To Sixth-Grade Low-Income Minority Children. J. Acad. Nutr. Diet..

[59] Barton B.A., Eldridge A.L., Thompson D., Affenito S.G., Striegel-Moore R.H., Franko D.L., Albertson A.M., Crockett S.J. (2005). The Relationship Of Breakfast And Cereal Consumption To Nutrient Intake And Body Mass Index: The National Heart, Lung, And Blood Institute Growth And Health Study. J. Am. Diet. Assoc..

[60] Gibson S.A., O’sullivan K.R. (1995). Breakfast Cereal Consumption Patterns And Nutrient Intakes Of British Schoolchildren. J. R. Soc. Health.

[61] Gibson S. (2003). Micronutrient Intakes, Micronutrient Status And Lipid Profiles Among Young People Consuming Different Amounts Of Breakfast Cereals: Further Analysis Of Data From The National Diet And Nutrition Survey Of Young People Aged 4 To 18 Years. Public Health Nutr..

[62] Ortega R.M., Requejo A.M., Redondo R., Lopez-Sobaler A.M., Andres P., Ortega A., Gaspar M.J., Quintas E., Navia B. (1996). Influence Of The Intake Of Fortified Breakfast Cereals On Dietary Habits And Nutritional Status Of Spanish Schoolchildren. Ann. Nutr. Metab..

[63] Ortega R.M., Requejo A.M., Lopez-Sobaler A.M., Andres P., Quintas M.E., Navia B., Izquierdo M., Rivas T. (1998). The Importance Of Breakfast In Meeting Daily Recommended Calcium Intake In A Group Of Schoolchildren. J. Am. Coll. Nutr..

[64] Mcnulty H., Eaton-Evans J., Cran G., Woulahan G., Boreham C., Savage J.M., Fletcher R., Strain J.J. (1996). Nutrient Intakes And Impact Of Fortified Breakfast Cereals In Schoolchildren. Arch. Dis. Child.

[65] Matthys C., De Henauw S., Bellemans M., De Maeyer M., De Backer G. (2007). Breakfast Habits Affect Overall Nutrient Profiles In Adolescents. Public Health Nutr..

[66] Papoutsou S., Briassoulis G., Hadjigeorgiou C., Savva S.C., Solea T., Hebestreit A., Pala V., Sieri S., Kourides Y., Kafatos A. (2014). The Combination Of Daily Breakfast Consumption And Optimal Breakfast Choices In Childhood Is An Important Public Health Message. Int. J. Food Sci. Nutr..

[67] Nasir M.T.M., Nurliyana A.R., Norimah A.K., Mohamed H.J.B.J., Tan S.Y., Appukutty M., Hopkins S., Thielecke F., Ong M.K., Ning C. (2017). Consumption Of Ready-To-Eat Cereals (Rtec) Among Malaysian Children And Association With Socio-Demographics And Nutrient Intakes-Findings From The Mybreakfast Study. Food Nutr. Res..

[68] Murakami K., Livingstone M.B.E., Fujiwara A., Sasaki S. (2018). Breakfast In Japan: Findings From The 2012 National Health And Nutrition Survey. Nutrients.

[69] Gibson S.A. (2000). Breakfast Cereal Consumption In Young Children: Associations With Non-Milk Extrinsic Sugars And Caries Experience: Further Analysis Of Data From The Uk National Diet And Nutrition Survey Of Children Aged 1.5–4.5 Years. Public Health Nutr..

[70] Deshmukh-Taskar P.R., O’neil C.E., Nicklas T.A., Yang S.J., Liu Y., Gustat J., Berenson G.S. (2009). Dietary Patterns Associated With Metabolic Syndrome, Sociodemographic And Lifestyle Factors In Young Adults: The Bogalusa Heart Study. Public Health Nutr..

[71] Castillo Valenzuela O., Liberona Zúñiga J., De Landa A.D., Thielecke F., Mondragón M.M., Narkunska J.R., Muñoz S.C. (2015). Consumption Of Ready-To-Eat Cereal Is Inversely Associated With Body Mass Index In 6–13 Years Old Chilean Schoolchildren. Nutr. Hosp..

[72] Kosti R.I., Panagiotakos D.B., Zampelas A. (2010). Ready-To-Eat Cereals And The Burden Of Obesity In The Context Of Their Nutritional Contribution: Are All Ready-To-Eat Cereals Equally Healthy? A Systematic Review. Nutr. Res. Rev..

[73] Priebe M.G., Mcmonagle J.R. (2016). Effects Of Ready-To-Eat-Cereals On Key Nutritional And Health Outcomes: A Systematic Review. PLoS ONE.

[74] Galvin M.A., Kiely M., Flynn A. (2003). Impact Of Ready-To-Eat Breakfast Cereal (Rtebc) Consumption On Adequacy Of Micronutrient Intakes And Compliance With Dietary Recommendations In Irish Adults. Public Health Nutr..

[75] Song W.O., Chun O.K., Kerver J., Cho S., Chung C.E., Chung S.J. (2006). Ready-To-Eat Breakfast Cereal Consumption Enhances Milk And Calcium Intake In The Us Population. J. Am. Diet. Assoc..

[76] Powers H.J., Stephens M., Russell J., Hill M.H. (2016). Fortified Breakfast Cereal Consumed Daily For 12 Wk Leads To A Significant Improvement In Micronutrient Intake And Micronutrient Status In Adolescent Girls: A Randomised Controlled Trial. Nutr. J..

[77] Utter J., Scragg R., Mhurchu C.N., Schaaf D. (2007). At-Home Breakfast Consumption Among New Zealand Children: Associations With Body Mass Index And Related Nutrition Behaviors. J. Am. Diet. Assoc..

[78] Baric I.C., Satalic Z. (2002). Breakfast Quality Differences Among Children And Adolescents In Croatia. Int. J. Food Sci. Nutr..

[79] Baric I.C., Satalic Z. (2003). Breakfast Food Patterns Among Urban And Rural Croatian Schoolchildren. Nutr. Health.

[80] Nicklas T.A. (2003). Calcium Intake Trends And Health Consequences From Childhood Through Adulthood. J. Am. Coll. Nutr..

[81] Tunick M.H., Van Hekken D.L. (2015). Dairy Products And Health: Recent Insights. J. Agric. Food Chem..

[82] Górska-Warsewicz H., Rejman K., Laskowski W., Czeczotko M. (2019). Milk And Dairy Products And Their Nutritional Contribution To The Average Polish Diet. Nutrients.

[83] Nicklas T.A., O’neil C.E., Berenson G.S. (1998). Nutrient Contribution Of Breakfast, Secular Trends, And The Role Of Ready-To-Eat Cereals: A Review Of Data From The Bogalusa Heart Study. Am. J. Clin. Nutr..

