# Supplementary material for: Breakfast Characteristics and Its Association with Daily Micronutrients Intake in Children and Adolescents–A Systematic Review and Meta-Analysis
Source: Nutrients. 2020 Oct 20;12(10):3201. doi: 10.3390/nu12103201 (PMC7589686; doi:10.3390/nu12103201)
Supplement: Supplementary file 1 [file nutrients-12-03201-s001.pdf]

AXIS tool: Quality Appraisal Findings

Key
Y = Yes
N = No/Not Reported

	Introduction	Methods										Results					Discussion		Other	
Author, Year	Clear Aims/ Objectives	Appropriate Study Design	Justified Sample Size	Defined Target Population	Appropriate Sampling Frame	Appropriate Sampling Method	Non-Respondents Addressed	Risk and Outcome Variables	Valid Data Collection Tools Used	Use of Statistical Significance and Precision	Study is	Adequate Data Description	Non-Response Bias Concerns	Description of Non-Respondents	Internal Consistency of Results	All Analyses Described	Conclusions Justified by	Limitations Reported	Funding/ Conflicts of Interest Declared	Ethics/Consent in place
Affenito, S. et al. 2005	Y	Y	Y	Y	Y	Y	N	Y	N	Y	Y	N	N	N	Y	Y	Y	Y	Y	Y
Affenito, S. et al. 2013	Y	Y	Y	Y	Y	Y	N	Y	N	Y	Y	Y	N	N	Y	Y	Y	Y	N	Y
Afeiche, M. et al. 2017	Y	Y	Y	Y	Y	Y	N	Y	N	Y	Y	Y	N	N	Y	Y	Y	Y	Y	Y
Albertson, A. et al. 2003	Y	Y	Y	Y	Y	Y	N	Y	Y	Y	Y	Y	N	N	Y	Y	Y	Y	N	N
Albertson, A. et al. 2008	Y	Y	Y	Y	Y	Y	N	Y	Y	Y	Y	N	N	N	Y	Y	Y	Y	Y	Y
Balvin Frantzen, L. et al. 2013	Y	Y	N	Y	N	N	N	Y	Y	Y	Y	Y	N	N	Y	Y	Y	Y	Y	Y
Barr, S. et al.	Y	Y	Y	Y	Y	Y	**Y**	Y	**N**	Y	Y	Y	Y	N	Y	Y	Y	Y	Y	Y

	Introduction	Methods										Results					Discussion		Other	
Author, Year	Clear Aims/ Objectives	Appropriate Study Design	Justified Sample Size	Defined Target Population	Appropriate Sampling Frame	Appropriate Sampling Method	Non-Respondents Addressed	Risk and Outcome Variables	Valid Data Collection Tools Used	Use of Statistical Significance and Precision	Study is	Adequate Data Description	Non-Response Bias Concerns	Description of Non-Respondents	Internal Consistency of Results	All Analyses Described	Conclusions Justified by	Limitations Reported	Funding/ Conflicts of Interest Declared	Ethics/Consent in place
2014																				
Barr, SI. et al. 2018	Y	Y	?	Y	Y	?	?	Y	N	Y	Y	Y	N	N	Y	Y	Y	Y	Y	Y
Barton, B. et al. 2005	Y	Y	Y	Y	Y	Y	N	Y	Y	Y	Y	Y	N	N	Y	Y	Y	N	Y	Y
Coulthard, j. et al. 2017	Y	Y	Y	Y	Y	Y	N	**Y**	**Y**	Y	Y	Y	N	N	Y	Y	Y	Y	N	Y
Deshmukh-Taskar, p. et al. 2010	Y	Y	Y	Y	Y	Y	N	Y	N	Y	Y	Y	N	N	Y	Y	Y	Y	Y	Y
Fayet-Moore, F. et al. 2016	Y	Y	Y	Y	Y	Y	N	Y	Y	Y	Y	Y	N	N	Y	Y	Y	Y	Y	Y
Fayet-Moore, F. 2017	Y	Y	Y	Y	Y	Y	N	N	N	Y	Y	N	N	N	Y	Y	Y	Y	Y	Y
Fulgoni, VL. et al. 2019	Y	Y	?	Y	Y	?	?	Y	N	Y	Y	Y	N	N	Y	Y	Y	Y	Y	Y
Gibson, S. et al. 1995	Y	Y	Y	Y	Y	Y	N	Y	Y	Y	Y	Y	N	N	Y	Y	Y	N	N	N

	Introduction	Methods										Results					Discussion		Other	
Author, Year	Clear Aims/ Objectives	Appropriate Study Design	Justified Sample Size	Defined Target Population	Appropriate Sampling Frame	Appropriate Sampling Method	Non-Respondents Addressed	Risk and Outcome Variables	Valid Data Collection Tools Used	Use of Statistical Significance and Precision	Study is	Adequate Data Description	Non-Response Bias Concerns	Description of Non-Respondents	Internal Consistency of Results	All Analyses Described	Conclusions Justified by	Limitations Reported	Funding/ Conflicts of Interest Declared	Ethics/Consent in place
Gibson, S. et al. 1999	Y	Y	Y	Y	Y	Y	N	Y	Y	Y	Y	N	N	N	Y	Y	Y	Y	Y	Y
Gibson, S. et al. 2003	Y	Y	Y	Y	Y	Y	N	Y	Y	Y	Y	N	N	N	Y	N	Y	N	Y	Y
Matthys, C. et al. 2007	Y	Y	N	Y	Y	N	N	Y	Y	Y	Y	Y	N	N	Y	Y	Y	Y	Y	Y
McNulty, H. et al. 1996	Y	Y	N	Y	N	N	N	Y	N	Y	Y	N	N	N	Y	Y	Y	N	Y	Y
Michels, N. et al. 2015	Y	Y	Y	Y	Y	Y	N	Y	N	Y	Y	Y	N	N	Y	Y	Y	Y	Y	Y
Mielgo-Ayuso, J. et al. 2017	Y	Y	Y	Y	Y	Y	N	Y	N	Y	Y	Y	N	N	Y	Y	Y	Y	N	Y
Mohd Nasir, MT. et al. 2017	Y	Y	Y	Y	N	N	N	Y	N	Y	Y	Y	N	N	Y	Y	Y	Y	Y	Y
Morgan, KJ. et al. 1981	Y	Y	N	Y	Y	Y	N	Y	N	Y	Y	Y	N	N	Y	Y	Y	N	N	N
Murakami, K.	Y	Y	Y	Y	N	N	N	Y	N	Y	Y	Y	N	N	Y	Y	Y	Y	N	Y

	Introduction	Methods										Results					Discussion		Other	
Author, Year	Clear Aims/ Objectives	Appropriate Study Design	Justified Sample Size	Defined Target Population	Appropriate Sampling Frame	Appropriate Sampling Method	Non-Respondents Addressed	Risk and Outcome Variables	Valid Data Collection Tools Used	Use of Statistical Significance and Precision	Study is	Adequate Data Description	Non-Response Bias Concerns	Description of Non-Respondents	Internal Consistency of Results	All Analyses Described	Conclusions Justified by	Limitations Reported	Funding/ Conflicts of Interest Declared	Ethics/Consent in place
et al. 2018																				
Ortega, RM. et al. 1996	Y	Y	N	Y	N	N	N	Y	Y	Y	Y	Y	N	N	Y	Y	Y	N	N	Y
Ortega, RM et al. 1998	Y	Y	N	Y	N	N	N	Y	Y	Y	Y	Y	N	N	Y	Y	Y	N	Y	Y
Papoutsou, S. et al. 2014	Y	Y	Y	Y	Y	Y	N	Y	N	Y	Y	Y	N	N	Y	Y	Y	Y	N	Y
Preziosi, P. et al. 1999	Y	Y	N	Y	Y	Y	N	Y	N	Y	Y	N	N	N	Y	Y	Y	N	N	N
Ramsay, SA. et al. 2018	Y	Y	N	Y	Y	Y	N	Y	N	Y	Y	Y	N	N	Y	Y	Y	Y	Y	Y
Ruxton, CH. et al. 1996	Y	Y	N	Y	Y	Y	N	Y	Y	Y	Y	N	N	N	Y	Y	Y	N	Y	Y
Vatanparast, H. et al. 2019	Y	Y	Y	Y	Y	Y	N	Y	N	Y	Y	Y	N	N	Y	Y	Y	Y	Y	Y
Williams, BM. et al. 2009	Y	Y	Y	Y	Y	Y	N	Y	N	Y	Y	Y	N	N	Y	Y	Y	Y	Y	N

	Introduction	Methods										Results					Discussion		Other	
Author, Year	Clear Aims/ Objectives	Appropriate Study Design	Justified Sample Size	Defined Target Population	Appropriate Sampling Frame	Appropriate Sampling Method	Non-Respondents Addressed	Risk and Outcome Variables	Valid Data Collection Tools Used	Use of Statistical Significance and Precision	Study is	Adequate Data Description	Non-Response Bias Concerns	Description of Non-Respondents	Internal Consistency of Results	All Analyses Described	Conclusions Justified by	Limitations Reported	Funding/ Conflicts of Interest Declared	Ethics/Consent in place
Williams P. et al. 2007	Y	Y	Y	Y	Y	Y	N	Y	N	Y	Y	Y	N	N	Y	Y	Y	N	N	Y
